# History of the Epidemic of Cholera at the Philadelphia Almshouse, Blockley, in the Summer of 1849

**Published:** 1849-11

**Authors:** Moreton Stillé, Edward R. Mayer

**Affiliations:** Late Medical Superintendant of the Cholera Hospital in that Institution; Late Medical Superintendant of the Cholera Hospital in that Institution


*History of the Epidemic of Cholera at the Philadelphia Almshouse, Blockley, in the Summer of* 1849. By Moreton Stillé, M. D., and Edward R. Mayer, M. D., late Medical Superintendants of the Cholera Hospital in that Institution.

The undersigned have prepared the following short history of the late epidemic, with the desire of contributing as far as they are able to the stock of facts now accumulating, relative to the Asiatic Cholera. The account which we have to offer is, in many respects, imperfect; the disease having prevailed already to a great extent, before we came upon duty. We have been furnished with a history of the cases which occurred before the opening of the cholera hospital, and will refer to them in a general view of the extent and mortality of the epidemic; but further than this we are not able, nor do we feel at liberty, to make use of them. We will, therefore, confine our observations to the cases which were registered upon the books of the cholera hospital, and which came directly under our charge. These books are, indeed, defective in some points, owing to the constant occupation of the medical attendants in the early part of the season. We believe, however, that the few omissions which exist, have been so far supplied by our own recollection of individual cases, as not materially to affect the value of the general result. The situation and general plan of the Almshouse are known to almost every one in this community. For the information of others, we would state, that the buildings, with an external measurement of probably 900 by 700 feet,) bound an extensive quadrangle, open at the four corners, and are situated upon rising ground on the right bank of the Schuylkill river. The large area enclosed by them is divided by high walls into several smaller courts, and in its centre is situated a two-storied stone building, the ground floor of which is open and is used as a wash-house. The four rooms on the second story were opened as a cholera hospital early in the month of July. This building was selected for the purpose, because it was equidistant from opposite parts of the quadrangle. It was considered that the objections to its appropriation for this use, arising from its position in an enclosed court yard, would not outweigh the advantages derived from the convenience of the situation in other respects. In consequence of the high rate of mortality which continued to prevail, the Medical Board, consisting of Drs. Benedict, Page, Clymer, and one of ourselves, recommended the erection of a temporary hospital on the Almshouse ground, outside of the enclosure. This measure was approved by the Board of Guardians, and directions were given for the construction of two frame buildings, of suitable size, for hospitals, with the necessary offices. The new buildings were, however, not ready for the reception of patients until the 23d of July, before which time the epidemic had already begun to decline; and, indeed, after the occupation of these buildings by the cholera patients, although the elevated site upon which they stood was incontestably salubrious, the previous high mortality of the disease continued unaffected. The removal of *all* the inmates of the institution into a temporary camp outside of the walls, was suggested to, and maturely considered by, the Board of Guardians, aided by the advice of some of the most eminent physicians of the city: the project was, however, deemed to be not advisable, in view of the number and character of the population of the house, and the advanced period of the epidemic. Indeed, had such a removal been practicable, within four weeks from the time of its suggestion, (which is altogether improbable,) the certain injury to the occupants of the hospitals, to the old, blind and infirm women and men, the incurables, lunatics and idiots would, we think, have swelled the list of deaths above the point to which the mortality from cholera raised it, and that too, with a very doubtful prospect of staying the ravages of the epidemic. This plan was therefore rejected, and the attention of the Medical Board and of the Board of Guardians, directed to the execution of those sanatory regulations which seemed most advisable. Barrels of pitch were burned within the enclosure, to create an upward current of air; fires were kindled in the wards, and the windows thrown open, in order thoroughly to ventilate the house; the courts were well cleansed, and the vicinity of the privies and the opening of the sewers, liberally covered with slacked lime. The Medical Board moreover suggested, that such persons as were physically able to leave the Almshouse should be allowed to depart, and be induced to do so by the offer of a weekly stipend for their maintenance elsewhere. Owing to the adoption and execution of this measure, a large number left the house. It was further recommended, that woollen under clothing should be freely distributed, the diet scale improved, and all those living upon the charity of the institution daily inspected by the resident physicians, in order that the disease might be checked in its incipient stages. These measures were not, however, carried out to the extent desired by the Board. That last mentioned was certainly no easy task, as the medical superintendent of the house was suffering from repeated attacks of illness, and the number of resident physicians on duty was reduced by sickness and absence, to five.

The care of all the cholera patients was, at the outbreak of the epidemic, and for several days afterwards, undertaken by the usual medical officers, but owing to the rapid spread of the disease, their duties became entirely too laborious, and upon the 7th day of July a separate cholera service was instituted, over which we were, in turn, appointed to preside. We received, during our attendance, the most valuable assistance from a number of gentlemen, graduates and students of medicine. With a devotion rendered the more praiseworthy from the fact of many of them being far from their homes and friends, they cheerfully offered their gratuitous assistance, and ministered with untiring assiduity to all the wants of the sick and dying.* The

*The following are the names of the gentlemen referred to: Mr. T. M. Flint, of Philadelphia, J. Warren White, Mississippi; David P. Heap, Tunis, Africa; Ptolemy S. Harris and Charles Heerman, Louisiana; Theophilus Field and Benjamin H. Walker, Virginia; George M. Darrach, Pa. The following having previously been on duty, were appointed the Assistant Resident Physicians of the new cholera hospital: Drs. Arthur M. Lynah, U. S. N.; Robt. C. Black, Ga.; Thomas S. Mills, Va.; John D. McCall, Lima, S. A.

health of some became materially prejudiced by the prevailing epidemic influence ; the lives of two were seriously threatened by the disease, while two others, Mr. Flint, of Philadelphia and Mr. White, of Mississippi, died in the city of cholera, a few days after they had been obliged, by ill-health, to give up their duties at the Hospital. The services which fell to the lot of ourselves and our medical assistants were, in consequence of the want of good nurses, numerous and trying.

The absence in Europe, of Dr. Clymer, the consulting physician of the Almshouse, renders it not improper that we should here make mention of his active and efficient co-operation in all our measures, and his zealous perseverance in them under the most disheartening circumstances, as well as acknowledge the benefits of his medical experience and research.

The nurses of the Cholera Hospital were for some time entirely taken from among the paupers, who were not only wholly incompetent, but moreover indolent and ill-disposed, and deserted us often at the most critical moment. Their place was afterwards supplied by hired men from the city.

Another serious annoyance was the necessary employment, during the early part of the epidemic, of the paupers, as carriers of the sick. The cases occurring in the Almshouse were sent for, as soon as they were reported, but owing to the supineness and delay upon the part of the carriers, several hours often elapsed before the patients were brought to us for treatment. It will be easily understood, that to this cause, may be very justly attributed a part of the unfavorable issue of the cases which came under our charge.

The epidemic commenced in the Almshouse on the 29th of July. Previous to this, four cases had been brought in from the city. Two of them were received on the 25th and 26th of July, and had been placed in the Women's Medical Ward, and the other two in the men's hospital. The first case that occurred in the institution was in the Women's Medical Ward, into which the cases from the city had been brought. The first case on the men's side was in the Men's Surgical Ward. We are unable to ascertain whether the cases from the city had been placed in this or in the medical ward above it. However, the disease seemed to spread from these two points, not step by step, but rapidly, to all other parts of the establishment. Without wishing to prejudge the question of the contagious nature of cholera, we would merely state, that such of the pauper population of the house as are physically able, are employed in all menial services, as far as they can be made useful in this way, and that they help to nurse the sick and carry messages from one part of the building to another. We were not able to trace any connexion between the prevalence of the cholera and the condition of the atmosphere. The barometer maintained a high average, the heat was by no means as great as it often is in our summer, and the direction of the wind and character of the weather presented no constant feature. The want of dependence of the cholera upon a paucity of electricity in the atmosphere, is shown by the fact that there were several thunderstorms during the epidemic, and that after a remarkably violent thundergust on the 14th of July, the number of cases *increased* daily for some time. A meteorological table appended to this article may be further consulted in reference to this subject.

The lunatic wards suffered more than any other. They furnished nearly one-third of the whole number of patients, and the disease, as it occurred in them, was peculiarly intractable. We are unable to assign any more plausible cause for the liability of the inmates of these wards to the disease, than the fact that nearly all were labouring under the more depressing forms of insanity, as melancholy, imbecility, or idiocy; their mental and moral sources of resistance were gone, while physically they were in the most unfavourable condition, as very few of them enjoyed the liberty of exercise in the open air, and their diet consisted almost exclusively of soup and vegetables, and was, consequently, far from being nutritious. A fact may be mentioned as affording some support to these views, viz., that of twenty lunatics employed by the gardener as assistants not one was attacked by the disease. On the other hand, their wards are roomy, scrupulously clean, and well ventilated. The "Lodge" in which the more violent patients are kept, is about fifty yards distant from the main building, and *entirely outside of the enclosure*, and although it is said to be somewhat damp, is clean, and has the benefit of a thorough draft of air. Yet in this building the cholera raged with peculiar severity, as fully one-half of its inmates were carried off by it. The wards of the hospital, and the infirmaries for the old and blind and incurable supplied two-fifths of the whole number of cases. The influence of other diseases or infirmities in predisposing to cholera, may be judged of from the fact that the " out-wards," containing the least feeble inmates of the Almshouse, furnished a far less number of patients in proportion to their population, than any other parts of the institution.

Thus it will be seen that two-thirds of all the persons treated in the cholera hospitals, came from wards, a residence in which alone implied circumstances predisposing to the disease and unfavourable to the power of resisting it. Previous sickness or imbecility, old age, intemperance and other vices, had already rendered them easy victims. Out of the whole number of persons whose age was recorded, viz., 161, nearly one-half were over fifty years of age, and three-fourths over forty. Many of the old people were habitual opium eaters, and all, invalids either from natural causes, or from actual disease or vice. The inmates of the medical and surgical wards were, of course, easily carried off by the disease, as they were already suffering from maladies mostly of a serious nature. In view of facts like these, we do not think it possible to institute any just comparison between the mortality of the epidemic in the Almshouse, and that of other places or institutions not similarly situated.

Besides these sources of the mortality, which our tables exhibit, a most prominent one is the fact, that from various causes, including the delays before referred to, the majority of patients were not placed under our care until they were already in the collapsed state. Fully nine-tenths of the patients transferred to us from the Almshouse, were in this condition. The only persons we had the good fortune to treat, from the commencement of their illness, were two of our medical assistants, two nurses, and another patient coming accidentally under our care. These all had well-marked symptoms of cholera, and recovered under prompt and active treatment. The other nurses of the cholera hospital whom we treated, neglected to report themselves until they were no longer able to attend to their duty, and the disease had advanced quite beyond the reach of medicine. Including the above, about twenty persons were under our treatment in the hospital before they had entered the perfectly collapsed state. These all had cramp, and vomiting, and purging of the characteristic liquids, and with the exception of one or two, were very strongly marked cases. Nearly all of these recovered perfectly.

The number of deaths among the convalescent patients was increased by the want of competent nurses in the female convalescent ward, and the imprudence of the patients themselves. In regard to the most important circumstances connected with cholera, peculiarities in its character and the comparative success of different plans of treatment, we are forced to own that we have but little to relate. We have reason to believe, without certainly knowing it to be the case, that the attack was in nearly all instances, ushered in by the usual diarrhœa. The patients were, however, generally presented to our notice at a period when the rice water liquids had ceased to be discharged externally to any amount.

The cyanosed hue, the cold and shrivelled skin, the irritable stomach, the whispering voice, muscular debility and mental apathy, the more prominent symptoms of the true collapse, were nearly always present upon their admission into the wards. In probably less than one-third of the cases were rice water evacuations either common or abundant, and cramps were still less frequently met with. In the medical management of the sick our best directed efforts proved constantly ineffectual, owing partly to the feeble vitality of the greater number, but chiefly, without any doubt, to the late period at which they were subjected to treatment. A fair trial was given to every remedy and every plan of treatment which had substantial experience in its favor, but we were constantly disappointed in our hopes, by finding many which had been the most highly vaunted, fail entirely in our hands. In the cases in which venesection could be practised, it was tried, but without success. Previous to the state of collapse, more benefit was derived from the administration of opium and acetate of lead, conjoined with sinapisms, laudanum enemata, and carefully regulated internal stimulation, than from any other treatment. Calomel in large doses, combined with opium, succeeded well in a few cases. When given in the manner proposed by Mr. Ayre, no good result was obtained. Quinine, in both large and small doses, astringent injections, ice frictions, warm and saline baths, venous injections and other popular means were frequently and fully made use of, but usually with little effect. Chloroform was not found to produce any more beneficial effect than that, in conjunction with other means, it generally relieved the vomiting and cramps when they existed, and enabled the patients to pass through their short term of life more comfortably than they would otherwise have done. A plan of treatment frequently employed in the cases of collapse, was the administration of a solution of camphor in chloroform, with the addition of kreasote, oil of turpentine and laudanum, conjoined with frictions of Lee's ointment modified,* and the use of the hot iron applied upon the spine over flannel previously moistened with Petit's embrocation of ammonia and turpentine. By these means patients were frequently roused from a state of collapse, again and again, and were then placed on the use of calomel and other remedies, but the restoration was nearly always but temporary, and the patient again relapsed into the condition which precedes death. Indeed, this mode of treatment was so often efficacious in bringing on speedy reaction, that we had every reason to believe that its use at an earlier period of the disease would have been eminently successful. But, under the circumstances which existed, we are called upon to record its almost constant failure, along with that of every other kind of practice used.

The tables which follow require some previous explanation. We have no positive information of any cases of cholera occurring in the Almshouse, except those registered upon our books. Besides these, we have a list of cases occurring before the opening of the Cholera Hospital on the 7th of July, which came under the care of the usual medical officers of the Almshouse. We have therefore divided the whole number of cases into two series, the first comprising those which occurred before the 7th of July; the second including those which were admitted into the Cholera Hospital until its closure, on the 4th of August. If reference be made to the bulletins of the Board of Health, considerable discrepancy will be found between the daily and weekly reports of cholera in the Almshouse, and between either of them and our own: we have endeavored, but in vain, to reconcile these varying results. We will vouch for the correctness of our own report, as far as it goes, and would remark at the same time, that we have entered upon our books several deaths which occurred in the convalescent ward, but which, through inadvertence, were not reported by us to the Board of Health. It is possible that other errors may have escaped our observation, but if it be

*℞. Camphoræ, lbj.

Chloroform, f℥viij.

Misce et adde

Capsici, lbss.

Ung. hydrarg. lbj.

so, it is nevertheless certain that they can have no sensible influence on the general result.

*Weekly Statement of the Population of the Almshouse during the Epidemic of Cholera.*

| June 30. | July 7. | July 14. | July 21. | July 28. | Aug. 4. |
|---|---|---|---|---|---|
| 1738 | 1691 | 1546 | 1358 | 1313 | 1305 |

FIRST SERIES.

*Cholera Cases occurring in the Almshouse before the 7th of July.*

| | *Male.* | *Female.* | *Total.* | *Black.* | *White.* | *Total.* |
|---|---|---|---|---|---|---|
| Cases, - | 29 | 24 | 53 | 14 | 39 | 53 |
| Deaths, - | 24 | 21 | 45 | 12 | 33 | 45 |
| Recoveries, | 5 | 3 | 8 or 15.09 per ct. | 2 | 6 | 8 |

Of these eight, two women died subsequently of adynamic fever, leaving

| | *Males.* | *Female.* | |
|---|---|---|---|
| Permanent recoveries, | 5 | 1 | 6 or 11.32 per cent. |

SECOND SERIES.

*Cases admitted into the Cholera Hospital between July 7th and August 4th.*

| | *Males.* | *Females.* | *Total.* | *Black.* | *White.* | *Total.* |
|---|---|---|---|---|---|---|
| Cases, - | 117 | 105 | 222 | 18 | 204 | 222 |
| Deaths, - | 99 | 84 | 183 | 16 | 167 | 183 |
| Recoveries, | 18 | 21 | 39 | 2 | 37 | 39 |

or 17.56 per cent.

Of these 39, there died subsequently of diarrhœa, dysentery, dynamic fever or relapses, six men and three women, leaving

| | *Males.* | *Females.* | |
|---|---|---|---|
| Permanent recoveries, 30, | 15 | 15 | or 13.51 per ct. |

Total number of Cholera cases recorded are—

| | *Males.* | *Females.* | *Total.* | *Black.* | *White.* | *Total.* |
|---|---|---|---|---|---|---|
| Cases, - | 146 | 129 | 275 | 32 | 243 | 275 |
| Deaths, - | 123 | 105 | 228 | 28 | 200 | 228 |
| Recoveries, | 23 | 24 | 47 | 4 | 43 | 47 |

| | | |
|---|---|---|
| Recovered from Cholera | 47 | or 17.09 per cent. |
| Died subsequently, | 11 | |
| Permanently recovered, | 36 | or 13.09 per cent. |

The *ages* of 161 patients in the Second Series are as follow; those of the First not having been recorded.

| | | | | |
|---|---|---|---|---|
| 4 patients from 1 to 10 years. | | 26 patients from 60 to 70 years. |
| 7 " " 10 to 20 " | | 17 " " 70 to 80 " |
| 15 " " 20 to 30 " | | 7 " " 80 to 90 " |
| 26 " " 30 to 40 " | | 3 " " 90 to 100 " |
| 31 " " 40 to 50 " | | 1 patient over 100 years. |
| 24 " " 50 to 60 " | | |

The wards or occupation of 247 patients, of both series, are as follow:

| | |
|---|---|
| From the Lunatic Wards, | 73 |
| " Blind, Old, Infirm and Incurable, | 54 |
| " Medical and Surgical, | 40 |
| " Ulcer and Venereal, | 4 |
| " Nursery and Obstetrical, | 7 |
| " Out Wards, | 41 |
| " City, | 5 |
| " Children's Asylum, | 4 |
| " Receiving Ward and Gate, | 4 |
| " Kitchen and Shoemaker's Shop, | 2 |
| Nurses of Cholera Hospital, | 6 |
| Nurses of Cholera Convalescent Ward, | 1 |
| Other Nurses, | 4 |
| Physicians, | 2 |
| | 247 |

The *place of birth* of 195 patients of the Second Series was recorded as follows:

| | *White.* | *Black.* | |
|---|---|---|---|
| From the United States, | 59 | 31 | 90 |
| " Great Britain, | | | 16 |
| " Ireland, | | | 65 |
| " Germany, | | | 21 |
| " Holland, | | | 1 |
| " France, | | | 1 |
| " South America, | | | 1 |
| | | | 195 |

*State of the Thermometer, Barometer, Dew Point, Wind and Weather, as observed daily, at 9, A. M., at the Pennsylvania Hospital, from the 20th of June to the 5th of August.*

| Date. | Thermom. | Dew Point. | Barom. | Winds. | Weather. |
|---|---|---|---|---|---|
| June, | | | | | |
| 20 | 81 | 63 | 30.23 | SW. NW. | Clear. |
| 21 | 87 | 64 | 30.21 | NNE. | " |
| 22 | 88 | 72 | 30.18 | SW. NW. | " |
| 23 | 88 | 67 | 30.05 | NW. | " |
| 24 | 80 | 55 | 29.98 | NE. SW. | Variable, thunder storm. |
| 25 | 79 | 65 | 29.87 | NW. | Clear. |
| 26 | 79 | 57 | 29.90 | NNW. | " |
| 27 | 77 | 54 | 30.01 | NE. SW. | " |
| 28 | 73 | 68 | 29.95 | SW. S. | Variable. |
| 29 | 71 | 67 | 29.80 | NW. | " |
| 30 | 75 | 67 | 29.95 | SW. | Clear. |
| July, | | | | | |
| 1 | 79 | 62 | 29.82 | N. | Variable. |
| 2 | 79 | 62 | 29.82 | NE. | Clear. |
| 3 | 67 | 49 | 30.20 | NE. | " |
| 4 | 67 | 53 | 30.28 | E. | " |
| 5 | 67 | 52 | 30.22 | E. | Variable. |
| 6 | 71 | 60 | 30.25 | SW. | " |
| 7 | 72 | 67 | 30.24 | SW. | " |
| 8 | 74 | 71 | 30.13 | SE. | " |
| 9 | 72 | 68 | 30.19 | SE. | " |
| 10 | 70 | 65 | 30.23 | NE. E. | " |
| 11 | 75 | 70 | 30.30 | SE. | " |
| 12 | 82 | 71 | 30.32 | SW. | Clear. |
| 13 | 88 | 70 | 30.22 | NW. | " |
| 14 | 88 | 70 | 30.00 | NW. | Clear, thunder storm. |
| 15 | 65 | — | 30.28 | NNE. | Clear. |
| 16 | 70 | 45 | 30.32 | NE. | " |
| 17 | 72 | 58 | 30.22 | SW. | " |
| 18 | 74 | 61 | 30.21 | SW. | " |
| 19 | 71 | 67 | 30.25 | SE. | Fog, clear. |
| 20 | 79 | 74 | 30.19 | SW. | Variable, thunder storm. |
| 21 | 77 | 73 | 29.87 | SW. | " thunder. |
| 22 | 75 | 60 | 30.00 | N. | Clear. |
| 23 | 74 | 63 | 30.18 | NE. | " |
| 24 | 73 | 63 | 30.33 | NE. | " |
| 25 | 69 | 62 | 30.28 | E. | Variable. |
| 26 | 79 | 74 | 30.08 | SE. | " |
| 27 | 76 | 65 | 30.14 | SW. | Clear. |
| 28 | 71 | 54 | 30.17 | NE. | Variable. |
| 29 | 73 | 57 | 30.13 | NE. | Clear. |
| 30 | 76 | 70 | 30.10 | SW. | Cloudy, clear. |
| 31 | 79 | 75 | 30.02 | SE. | " " |
| Aug. | | | | | |
| 1 | 68 | 56 | 30.15 | NE. | Variable. |
| 2 | 71 | 59 | 30.26 | NW. | Clear. |
| 3 | 72 | 61 | 30.24 | SE. | " |
| 4 | 73 | 60 | 30.14 | SSW. | " |
| 5 | 77 | 70 | 30.10 | SW. | Variable. |

Below is exhibited the mean temperature, &c., for the months of July, 1848, and 1849.

JULY, 1848.

| | | | |
|---|---|---|---|
| Mean temperature, | | - - - - | 74.82°. |
| " at 9, A. M. | | - - - - | 74.74°. |
| Maximum of Thermometer, | (26th,) | - | 91°. |
| Minimum " | (17th,) | - | 59°. |
| Range " | | - - - | 32°. |
| Mean of diurnal variations, | | - - | 13.25°. |

JULY, 1849.

| | | | |
|---|---|---|---|
| Mean temperature, | | - - - - | 74.66°. |
| " at 9, A. M. | | - - - - | 74.18°. |
| Maximum of Thermometer, | (13th,) | - | 95°. |
| Minimum " | (4th,) | - | 59°. |
| Range " | | - - - | 36°. |
| Mean of diurnal variations, | | - - | 14.88°. |

